# Entropy Measures for Data Analysis II: Theory, Algorithms and Applications

**DOI:** 10.3390/e23111496

**Published:** 2021-11-12

**Authors:** Karsten Keller

**Affiliations:** Institut für Mathematik, Universität zu Lübeck, D-23562 Lübeck, Germany; keller@math.uni-luebeck.de

Entropies and entropy-like quantities are playing an increasing role in modern non-linear data analysis. Fields of their application reach from diagnostics in physiology—for instance, electroencephalography (EEG), magnetoencephalography (MEG), and electrocardiography (ECG)—to econophysics and engineering. During the last few years, classical concepts as approximate entropy and sample entropy have been supplemented by new entropy measures, such as permutation entropy and variants of it. Recent developments are focused on multidimensional generalizations of the concepts with a special emphasis on the quantification of coupling between time series and system components behind them. Some of the main future challenges in the field are a better understanding of the nature of the various entropy measures and their relationships, with the aim of their adequate application, including good parameter choices. The utilization of entropy measures as features in automatic learning and their application to large and complex data for classification, discrimination, and finding structural changes requires fast and well-founded algorithms.

This Special Issue extends a collection of contributions related to entropy measures given in [1]. It provides five papers addressing entropy and entropy-like concepts and their applications in a broader sense.

Paper [2] is concerned with discrete time series. Here, the range of a time series is a finite set on a categorical or ordinal level. The absence of moments in models for data of such a level requires using other characteristics such as, for example, entropies. The paper’s author introduces the concept of an RS-DARMA model, extending the concept of a discrete autoregressive moving-average process. RS stands for regime-switching as a special data-driven mechanism. The idea is that the range of a time series is divided into subranges, each being responsible for some specification in the next step. The case with only one subrange provides the usual discrete autoregressive moving-average process. The new model class obtained, for illustration, is applied to a nominal DNA sequence and an ordinal time series of cloudiness states. Special emphasis is given to the case of an RS-DAR(1) model, i.e., to an RS-DARMA model with a missing moving-average part and an AR part of the order of 1.

The second paper, [3], discusses the problem of lossy data compression. Here, in contrast to lossless compression, the constraint that the storage capacity can be too little for preserving all information considered. For this, the message importance of data is included in the discussion, and an importance-weighted reconstruction error is defined. The authors consider the optimization problem of minimizing this error depending on the available storage size and develop an optimal storage-allocation strategy. The concepts used for this are rather similar to entropy concepts. It is shown that data with highly clustered information importance favor high compression, but data with uniformly distributed information importance are incompressible.

The main objective of [4] is to study a special image-encryption scheme introduced in a former paper by the authors. This scheme preserves the input image statistics described by the auto-correlation matrix and can be combined with the JPEG compression standard. The considered compression stage is based on classical methods such as Discrete Cosine Transform and entropy coding. The proposed scheme is represented in the form of a mathematical model. On this basis, both compression and encryption processes are analyzed. The authors add some experimental studies to discuss the scheme’s effectiveness from a more practical viewpoint.

A further paper, [5], discusses a novel fault-identification method for rolling bearings, which combines four different methods. These are a variational mode decomposition (VMD), a method called average refined composite multiscale dispersion entropy (ARCMD), support vector machines (SVM), and a sophisticated optimization algorithm called LCPGWO. The here-used entropy-related ARCMDE proposed for extracting multiscale fault features is a modification of the dispersion entropy addressing single scales. The performance of the proposed method was compared with that of four other methods given in the literature with the result of having a smaller error, better stability, and higher reliability.

The last paper of this Special Issue, [6], is devoted to generalizing the concept of ordinal patterns and characterizing the order type of delay vectors in dynamic systems. Here, the authors note that the idea of using distributions of ordinal patterns for quantifying the complexity of time series and systems underlying them has been established as a successful method. As ordinal patterns are built upon the binary relation defining the usual order on the real numbers, the basic building block of a generalized ordinal pattern is a binary relation of the two-dimensional real space or, equivalently, a partition of it in two pieces. In this paper, some relatively general statements on conditions under which such a partition provides a sufficiently high separation potential for determining the Kolmogorov–Sinai entropy of a measure-preserving dynamic system are given. The paper also sheds some light on the success of the original concept of ordinal patterns.
